# Lack of association between serotonin transporter gene polymorphism 5-*HTTLPR *and smoking among Polish population: a case-control study

**DOI:** 10.1186/1471-2350-9-76

**Published:** 2008-08-08

**Authors:** Alicja Sieminska, Krzysztof Buczkowski, Ewa Jassem, Ewa Tkacz

**Affiliations:** 1 Department of Allergology, Medical University of Gdansk, ul. Debinki 7, 80-952 Gdansk, Poland; 2 Department of Family Medicine, Nicolaus Copernicus University in Torun, Collegium Medicum in Bydgoszcz, ul. Sklodowskiej-Curie 9, 85-094 Bydgoszcz, Poland; 3 Department of Pneumonology, Medical University of Gdansk, ul. Debinki 7, 80-952 Gdansk, Poland

## Abstract

**Background:**

A better understanding of the genetic determinants of tobacco smoking might help in developing more effective cessation therapies, tailored to smokers' genotype. Insertion/deletion polymorphism in the promoter region of the serotonin transporter gene (*5-HTTLPR*) has been linked to vulnerability to smoking and ability to quit. We aimed to determine whether *5-HTTLPR *genotype is associated with smoking behavior in Caucasians from Northern Poland and to investigate other risk factors for tobacco smoking.

**Methods:**

*5-HTTLPR *genotypes were determined in 149 ever smokers (66 females; mean age 53.0 years) and 158 gender and ethnicity matched never smoking controls (79 females; mean age 45.0 years) to evaluate the association of this polymorphism with ever smoking status. Analysis of smokers was performed to evaluate the role of *5-HTTLPR *in the age of starting regular smoking, the number of cigarettes smoked daily, pack-years, FTND score, duration of smoking, and the mean length of the longest abstinence on quitting. Genotype was classified according to the presence or absence of the short (*S*) allele vs. the long (*L*) allele of *5-HTTLPR *(i.e., *S/S *+ *S/L *vs. *L/L*). Logistic regression analysis was also used to evaluate correlation between ever smoking and several selected variables.

**Results:**

We found no significant differences in the rates of *S *allele carriers in ever smokers and never smokers, and no relationship was observed between any quantitative measures of smoking and the polymorphism. Multivariate analysis demonstrated significant association between the older age (OR = 4.03; 95% CI: 2.33–6.99) and alcohol dependence (OR = 10.23; 95% CI: 2.09–50.18) and smoking.

**Conclusion:**

*5-HTTLPR *seems to be not a major factor determining cigarette smoking in Poles. Probably, the risk of smoking results from a large number of genes, each contributing a small part of the overall risk, while numerous non-genetic factors might strongly influence these genetic undergrounds of susceptibility to smoking.

## Background

Tobacco smoking is the commonest addiction to psychoactive substances and, being an extremely harmful behavior, grossly compounds to general morbidity and mortality. Growing evidence from classic twin studies supports the significant influence of genetic factors in smoking initiation and persistence, as well as the ability to quit smoking [1,2]. Currently, studies focus on investigating candidate genes to gain a better understanding of a molecular basis for smoking behavior [3]. However, the precise contribution of individual genes in such a complex behavior remains uncertain. It is most likely that many genes influence it, including those that are involved in neurotransmitter pathways and nicotine metabolism [4].

Studies indicate that nicotine increases serotonin release in the brain, while nicotine withdrawal has an opposite effect [5,6]. It was hypothesized that smoking habits may be associated with diminished serotonin (5-HT) neurotransmission determined by genetic polymorphism [7]. The human serotonin transporter gene (*5-HTT*), being involved in serotonin reuptake, has appeared as a plausible candidate gene for susceptibility to smoking, the more so since its link with psychological traits relevant to smoking behavior was demonstrated [8,9], as well as with alcohol dependence, which increases the risk of tobacco smoking [10,11]. A 44-bp insertion/deletion polymorphism within the promoter region (*5-HTTLPR*) has been identified with two allelic variants, the long (*L*) and the short (*S*) form, which affect the transcriptional efficiency of the *5-HTT *gene [12]. Lerman et al. [7] first hypothesized that *S *allele might exert a protective effect against smoking and evaluated the association of smoking practices and smoking cessation with *5-HTTLPR*. However, they did not find any significant differences in the distribution of *5-HTT *genotypes between smokers and non-smokers in either Caucasian or African Americans. One of the possible explanations for the negative results of this initial case-control study could be the relatively small sample size (268 current smokers vs. 230 never smokers), which could decrease the statistical power to detect small gene effect. However, in contrast to these results, Ishikawa et al. [13] found in a Japanese sample of a similar size that individuals with S/S genotype were less inclined to smoke and/or could more easily stop smoking than others. Results of subsequent studies, including larger sample studies of over one thousand subjects [14], which attempted to replicate these findings [14-17], have not been consistent, which might result from different ethnic backgrounds being associated with different degrees of linkage disequilibrium with other genes loci [18]. Thus, the hypothesized association between the low-activity *S *allele and smoking remains controversial, although a recent positron emission tomography study provided new evidence of this possible link [19]. Therefore, there is the need for further replication of association studies to define the role of the *5-HTTLPR *in vulnerability to smoking.

Since there have been no investigations into the association of *5-HTTLPR *and smoking carried out so far in a Polish population, we conducted the present study. The purpose was to determine whether *5HTTLPR *genotype is associated with smoking behavior in Caucasians from Northern Poland, as well as to investigate other risk factors for tobacco smoking.

## Materials and methods

### The study sample and measures

The study sample was completed among patients and staff of the Academic Clinical Center in Gdansk and outpatients of the Department of Family Medicine, the Nicolaus Copernicus University of Torun and Collegium Medicum in Bydgoszcz (NCUT-CMB). They were asked to complete a questionnaire referring to socio-demographic data (age, gender, educational level) and categorical definitions of smoking status. Never smoker was someone, who either had never smoked at all or had never been daily smoker and had smoked less than 100 cigarettes (or the equivalent amount of tobacco) in his lifetime [20]. Ever smokers were defined as individuals who had smoked at least 100 cigarettes in their lifetime [20]. Current smokers were defined as individuals who, at the time of the survey, smoked cigarettes either daily or occasionally [20]. Former smokers were defined as those who had quit smoking at least 1 year before the study. To verify recent non-smoking status, the measurement of carbon oxide in exhaled air was performed in former smokers with the use of *Micro CO *smokelyser (Bedfont Instruments, Kent, UK). The level of education was recorded as primary, vocational, high and university. Three hundred and ten adult subjects, including 150 ever smokers (cases) and 160 gender matched never smoking controls, were recruited. All of them were Caucasians from the North of Poland.

Several quantitative measures of smoking behavior were also completed with the use of the questionnaire. They included: age of starting regular smoking, number of cigarettes smoked, and number of years of smoking. Pack-years were calculated using the average number of cigarettes smoked daily and the number of years smoked. Current daily smokers were also asked to complete the Fagerstrom Test for Nicotine Dependence [21] and give the longest abstinence period on quitting attempt. All reported periods of maximal abstinence were further calculated into days.

In addition, information on recent or prior treatment due to any psychiatric disorders (diagnosis and medications) and on alcohol dependence was obtained from respondents by self-report.

From all participants of the study, 8-mm of venous blood sample was collected into heparinized tubes. Samples were frozen and stored at -80°C until required for molecular genotypic analyses.

The institutional research ethics committees at the Medical University of Gdansk and the Nicolaus Copernicus University of Torun approved all study procedures, and all subjects provided written, informed consent prior to participation in the study.

### Genotyping

Genomic DNA was extracted from lymphocytes by an enzymatic method using a commercial kit *Blood DNA Prep Plus *(A&A Biotechnology, Gdynia) and used as a template for the PCR. *5-HTTLPR *genotyping was performed with the use of oligonucleotide primers flanking the *5-HTTLPR *as described by Heils et al. [12], and with a few modifications [22]. The set of primers used was as follows:

sense: 5'-GGCGTTGCCGCTCTGAATGC-3';

antisense: 5'-GAGGGACTGAGCTGGACAACCAC-3'.

### Statistical analyses

The chi-squared (χ^2^) test was used to asses the deviations of genotype distribution from the Hardy-Weinberg equilibrium and for group comparisons of frequencies of allele and genotype. Logistic regression analysis, using STATISTICA 7.1 software (StatSoft Inc., USA), was used to estimate correlations. Variables which appeared to be associated with any increased risk for smoking status in the univariate analysis were analyzed by multivariate analysis. The association between these variables and smoking was expressed as crude and adjusted odds ratios (Ors) with 95% confidence intervals (95% CIs). Student's test t was used to compare means for continuous variables. Because of the non-normal distribution of most parameters, nonparametric Mann-Whitney U test was applied for two-group comparisons. Data have been expressed as means ± standard deviation (SD). A significance level of 0.05 was set for a type 1 error in all analyses.

## Results

Three participants did not undergo genotyping successfully; they were, therefore, excluded from further investigation. As a result, the final sample for analysis consisted of 307 subjects, including 149 ever smokers (66 females; mean age 53.0 ± 11.2 years) and 158 never smokers (79 females; mean age 45.0 ± 16.2 years).

Distribution of *5-HTTLPR *genotypes did not deviate significantly from the Hardy-Weinberg expectation, as determined by the chi-square test (χ^2 ^= 0.80, df = 1; p = 0.37)

With carriers defined as subjects who tested positive for the presence of the allelic variants, whether homozygous or heterozygous (i.e., *S/S*, *S/L*), we found that 58% of the sample (n = 178) carried *S *allele (n = 42 homozygous), while 42% (n = 129) did not (i.e., *L/L*). Frequencies of *L *and *S *allele, as well as short allele carriers and non-carriers, did not differ significantly in ever and never smokers (χ^2 ^= 1.71, df = 1; p = 0.19, and χ^2 ^= 1.03; df = 1; p = 0.31, respectively). Similarly, there were no significant difference in the distribution of *5-HTTLPR L *and *S *alleles and genotypes in current and never smokers, as well as in current and former smokers. Allele frequencies and genotypes for the *5-HTT *gene by smoking status can be found in Table 1.

**Table 1:** Distribution of *5-HTTLPR L *and *S*eles and genotypes according to smoking status in the surveyed population from the North of Poland

Smoking status	No. (%) of allele		No. (%) of genotype		
	
	*L*	*S*	*L/L*	*L/S*	*S/S*
Ever smokers	199 (66.8)	99 (33.2)	67 (45)	65 (43.6)	17 (11.4)
Current smokers	131 (66.2)	67 (33.8)	43 (43.4)	45 (45.5)	11 (11.1)
Former smokers	68 (68)	32 (32)	24 (48)	20 (40)	6 (12)
Never smokers	195 (61.7)	121 (38.3)	62 (39.2)	71 (44.9)	25 (15.8)

In the smokers group, no association was observed between any quantitative measures of smoking and the polymorphism (Table 2).

**Table 2:** Values for smoking characteristics by 5-*HTTLPR *genotype in ever smokers

Characteristic	Mean (SD)		*P *value
		
	*S/S*+*S/L*	*L/L*	
Duration of smoking	27.4 ± 11.9	26.9 ± 12.7	0.76
No. of cigarettes smoked daily	19.6 ± 10.5	18.2 ± 8.9	0.58
No of pack/years	27.3 ± 20.0	22.4 ± 12.8	0.32
Age of proceeding to regular smoking	19.9 ± 4.5	20.5 ± 5.3	0.76
FTND score*	5.5 ± 2.5	5.3 ± 2.0	0.66
Duration of the longest abstinence in quitting attempts*	368.6 ± 1102.3	211.6 ± 388.2	0.90

*only current daily smokers were analysed

Out of the total of 307 participants, 23 subjects (7.5%; 15 females) reported current or prior treatment because of psychiatric disorders, including depression or anxiety-related disorders (20 subjects) and schizophrenia (3 subjects). Cited medications were in agreement with self-reported psychiatric diagnoses and included inhibitors of selective serotonine transporter (15 subjects), tricyclic antidepressants (4 subjects), neuroleptics (3 subjects), and anxiolytics (1 subject). There were 6 (26%) never smokers and 17 (74%) current smokers, while there were no former smokers among them. The rate of ever smokers among subjects reporting mental health problems was significantly higher than in other subjects (74% vs. 46.5%; p = 0.011). Seventeen subjects (5.5%; 3 females) in the study group mentioned alcohol dependence in the self-report. There were 15 ever smokers (88%) and two never smokers among them (12%). The frequency of smoking in alcoholics was significantly higher in comparison to non-alcoholic subjects (88% vs. 46%; p = 0.0018). We found no differences in frequencies of *S *and *L *alleles between subjects with psychiatric disorders or alcohol dependence and remaining subjects (p = 0.64 and p = 0.91, respectively).

In univariate logistic regression analyses, the following variables appeared to be associated with an increased risk for ever smoking status (i.e., smoking initiation): older age, a history of psychiatric disorders and alcohol dependence. Gender was not considered in these analyses because of the gender-matching of the study sample.

Multivariate regression analysis demonstrated a significant association between the older age and alcohol dependence and smoking, while adjusted odds ratio for smoking for subjects with psychiatric disorders nearly reached statistical significance (Table 3).

**Table 3:** Multivariate analysis of the association between ever tobacco smoking and selected variables

Variables	Crude ORs (95% CI)	Adjusted ORs (95% CI)*
*S/S *+ *S/L *genotype	0.79 (0.50–1.25)	0.78 (0.46–1.33)
Age ≥ 50 years	3.13 (1.96–5.00)	4.03 (2.33–6.99)
Lower education (primary/vocational)	1.36 (0.82–2.24)	0.98 (0.54–1.79)
Psychiatric disorder +	3.26 (1.25–8.55)	2.90 (0.98–8.55)
Alcohol dependence +	8.73 (1.95–39.13)	10.23 (2.09–50.18)

*Adjusted ORs are adjusted for all other items in the multivariate model

The results of the separate analyses on the association between *5-HTTLPR *and smoking, performed in more homogenous groups of ever and never smokers obtained by excluding subjects with psychiatric disorders and/or alcohol dependence, did not differ significantly from results of analyses performed in the whole study sample. Frequencies of *L *and *S *allele, as well as short allele carriers and non-carriers did not still differ significantly in ever and never smokers (χ^2 ^= 1.87, df = 1, p = 0.17, and χ^2 ^= 1.43, df = 1, p = 0.23, respectively). Similarly, we consistently found no association between any quantitative measures of smoking and the polymorphism in the smokers group (Table 4). Further, the results of multivariate analysis of the association between ever tobacco smoking and *5-HTTLPR*, age, and the level of education (adjusted ORs and 95% CI: 0.76, 0.43–1.34; 4.72, 2.63–8.46; 1.07, 0.57–2.01, respectively) were consistent with those when it was performed in the whole study sample.

**Table 4:** Values for smoking characteristics by 5-*HTTLPR *genotype in ever smokers after excluding subjects with self-reported psychiatric disorders and/or alcohol dependence

Characteristic	Mean (SD)		*P *value
		
	*S/S*+*S/L*	*L/L*	
Duration of smoking	24.8 ± 11.0	28.0 ± 13.1	0.18
No. of cigarettes smoked daily	18.0 ± 9.2	17.1 ± 8.5	0.73
No of pack/years	22.9 ± 15.0	16.1 ± 17.4	0.73
Age of proceeding to regular smoking	20.6 ± 4.3	20.5 ± 4.7	0.76
FTND score*	5.1 ± 2.4	5.2 ± 2.0	0.79
Duration of the longest abstinence in quitting attempts*	413.9 ± 1482.3	265.9 ± 446.4	0.29

*only current daily smokers were analysed

## Discussion

In the surveyed population from the North of Poland, we found 36% of carriers of the *5-HTTLPR *short variant allele. This rate was approximate to 34% found by another Polish research group [23], somewhat lower than those reported in two studies conducted in European Americans, where *S *allele was found in 40% and 43% of subjects, respectively, and somewhat higher than the rates found in African American participants of these studies, i.e., 30% and 31%, respectively [7,24]. In turn, the considerably lower rate of *S *allele was found in two studies conducted in Japanese populations – 16% and 19% [13,24]. These differences between *5-HTTLPR *allele frequencies in various populations reflect their racial and ethnic genetic differentiation [24].

We found no association between *5-HTTLPR *and smoking status, as well as any quantitative measure of smoking, such as the number of cigarettes smoked daily, the number of pack-years, FTND score, or the duration of the longest abstinence period on quitting. To date, only a few studies, in contrast to our findings, supported a link between *5-HTTLPR *and smoking, and demonstrated that individuals with the *L *allele were more inclined to smoke and/or had more difficulties with quitting smoking than others [13,15]. Other studies failed to replicate these positive results, including the more recent report of Trummer et al., who found additionally that neither smoking status nor Fagerstrom Tolerance Questionnaire score, pack-years, number of cigarettes smoked daily or previous attempts to quit smoking were related to *5-HTT *genotypes [7,14]. It should be noted, however, that several factors may have a substantial impact on the outcome of association studies and contribute to their inconsistency [3,18]. The most important is population heterogeneity, as regards ethnicity, gender or age, and possible stratification. Further, interacting effects, such as environment and personality, are considered to play an important role [25]. Between-study heterogeneity results also from the various categorical definitions of never smokers, current smokers and former smokers adopted in particular studies, as well as from differences in the mean number of cigarettes smoked per day or mean values of other quantitative measures of smoking behavior in smokers used in studies. If we compare, for instance, our survey to the Israeli study [15] which reported a highly significant association between *5-HTTLPR *genotype and smoking behavior, irrespective of dependence level, several essential differences regarding both populations might be found. Apart from population ancestry, the younger age of the participants of the Israeli survey seems to be one of them, with the average age of ever smokers being 29 years. In addition, smokers in this study group were mainly light smokers, not biologically dependent on nicotine (i.e., they had a FTND score lower than 6), while, in our study population, half of current smokers were highly addicted subjects. It is likely that these differences could influence the results of these two studies. Moreover, probably the effect of polymorphism is related to socio-cultural settings and ethnicity and may be less marked in populations of such countries like Poland, where smoking was a "national habit" for decades [26]. Ever smokers in our material were recruited mostly from individuals who had started smoking at a particular socio-political period of Polish history, which influenced the style of life of Poles with an extremely high consumption of cigarettes [26]. We found that subjects aged 50 years or older had an over three times higher risk of smoking than younger subjects. It is possible that possession of *S *allele, hypothesized to increase dopamine availability in midbrain, was not enough to protect from smoking in such a disadvantageous environment. Thus, non-replication of association studies may result from a small effect of a single gene and the relatively greater influence of a number of environmental factors on smoking. The link of the *5-HTTLPR *or other candidate gene polymorphism with smoking might emerge in a given population when smoking rates decline due to the predominance of non-genetic factors, including positive changes in the attitudes to smoking of the population as a whole.

It appears essential in case-control genetic association studies to precise defining of smoking phenotypes. The method of classification of ever smokers may have an impact on the results of the study [27]. It is suggested, that a significant amount of information on the resistance to regular smoking might be emerged with defining individuals who have smoked 1–99 cigarettes in their lifetime as ever smokers, not never smokers according to WHO [18,27]. It is also suggested, that genuine never smokers, who had never smoked even one cigarette (or even one puff) may be highly genetically informative [3]. However, in our study conducted in the population of extremely high prevalence of smoking by decades, subjects having smoked up to 100 cigarettes in their lifetime were considered never smokers.

Because of the divergent results of association studies, evidence for a substantial role of the *5-HTT *gene in smoking behavior is not strong to date. Additionally, meta-analysis of studies on association between the *5-HTTLPR *and smoking behavior [3] did not suggest an effect of this polymorphism on initiating, adopting and persisting with smoking. However, when only the studies which reported data on the *5-HTTLPR *polymorphism that pertained to smoking cessation were analyzed, a significant effect was revealed. It is likely that the presence of a variant allele may be associated with an increased likelihood of successful smoking cessation. In our study, the comparison of the mean length of the self-reported longest abstinence on quitting did not reveal significant differences between *S *allele carriers and non-carriers, which indirectly indicated that ability to quit might not be related to the *5-HTTLPR *genotype. Other genes, non-genetic factors, personality and motivation to quit might contribute a greater effect in smoking cessation.

Growing evidence indicates that smoking behavior and ability to quit are influenced by personality traits, including neuroticism, and psychiatric disorders or alcohol dependence [28,29]. On the other hand, several studies demonstrated the association between the *5-HTTLPR *and alcohol dependence or neuroticism, as well as depression and anxiety, which correlate well with this personality trait [10,11,30,31]. Data suggested also that smoking behavior is influenced by an interaction between neuroticism and *5-HTTLPR *genotype [32]. More recently, however, a number of studies demonstrated that although neuroticism and depression vulnerability were associated with smoking behavior, genotype did not affect this relationship [33,34].

Undergoing treatment for alcohol addiction and/or the presence of psychiatric disorders was an exclusion criterion imposed on subjects' eligibility for some of the earlier association studies on *5-HTTLPR *to avoid a possible confounding effect on the distribution of genotypes in smokers and non-smokers [7,13]. In the present study, this exclusion criterion was not adopted, although for example the results of other Polish researchers indicated an importance of careful inclusion of probands in studies on association between *5-HTTLPR *and personality traits [22,35], which in turn may affect smoking status. The fact that we found no differences in the frequencies of *S *and *L *alleles between subjects with psychiatric disorders or alcohol dependence and remaining subjects might suggest the lack of associations between psychiatric disorders and alcohol dependence and *5-HTT *genotype. However, the group size of subjects reporting these problems seems too small to generalize the results of this study, especially as regards the link of *5-HTTLPR *with separate psychiatric diagnoses. In several studies which focused on the investigation of a possible link of 5-*HTTLPR *with affective disorders, a positive association was found [30,31], while other studies have not supported this [36-38]. Thus, the relationship between this polymorphism and affective disorders remains uncertain. Our study let us only support earlier observations that individuals with psychiatric disorders and alcohol dependence are more likely to smoke cigarettes [29].

Several limitations of the study should be pointed out. Firstly, the study sample was relatively small, while sample size in thousands of subjects might be more sufficient to detect small genetic effects, which are likely for single loci and complex smoking behavior. However, several studies on the link of *5-HTTLPR *with smoking have been recently conducted in smaller samples, which included several hundreds of subjects, and positive association was reported [15-17]. This incited us to investigate the potential association between *5-HTTLPR *and smoking in the sample of a similar size in Polish population. Secondly, we have not genotyped a SNP within the *5-HTTLPR *[39], which could modify the effect of the *L *allele. Further, the smoking status has been verified with the measurement of exhaled CO concentration only in former smokers (i.e., those smokers who had quit smoking at least 1 year before the study) to avoid the classification error attributed to self-report [40]. In turn, we did not expect this bias toward a socially desirable response in never smokers and current smokers, therefore biochemical verification of smoking status was not performed in these groups. Finally, participants' self-reports of their psychiatric disorders were not confirmed by a formal clinical interview or checking medical documentation, but only confronted with cited pharmacological treatment. However, the main purpose of the present study was to assess the relationship between *5-HTTLPR *and smoking, and psychiatric disorders in general, as well as alcohol dependence, served only as covariates in our analyses.

## Conclusion

In spite of potential limitations, the results of our study allow us to conclude that *5-HTTLPR *is not a major factor determining cigarette smoking in Poles. Probably, the risk of smoking results from a large number of genes, each contributing a small part of the overall risk, while numerous non-genetic factors might strongly influence these genetic undergrounds of susceptibility to smoking. A better understanding of the genetic determinants of smoking needs further investigation into the interactions of genes involved in synthesis, release, uptake and receptor function for a variety of neurotransmitters, as well as into establishing the interactions between the *5-HTTLPR *polymorphism and psychological traits.

## Competing interests

The authors declare that they have no competing interests.

## Authors' contributions

AS conceived the study, participated in its design and coordination, performed the statistical analysis and drafted the manuscript. KB participated in the data collection phase, helped to interpret findings and contributed to the text. EJ participated in the design, coordination and supervision of the study and helped to draft the manuscript. ET participated in the data collection phase. All authors reviewed drafts of the manuscript and approved the final version before submitting it for publication.

## Pre-publication history

The pre-publication history for this paper can be accessed here:


